# The complete mitochondrial genome of the blue-crested lizard, *Calotes mystaceus* (Squamata, Agamidae) in China

**DOI:** 10.1080/23802359.2020.1822219

**Published:** 2020-10-07

**Authors:** Mo Wang, Zhuoyun Jiang, Jishan Wang, Lin Cui, Mingwang Zhang

**Affiliations:** a Key Laboratory for Conserving Wildlife with Small Populations in Yunnan, Faculty of Biodiversity Conservation, Southwest Forestry University, Kunming, China; b State Key Laboratory of Genetic Resources and Evolution, Kunming Institute of Zoology, Chinese Academy of Sciences, Kunming, China; c China Forest Exploration & Design Institute in Kunming, State Forestry and Grassland Administration P.R. China, Yunnan, China; d College of Animal Science and Technology, Sichuan Agricultural University, Chengdu, PR China; e Animal Genetic Resources Exploration and Innovation Key Laboratory of Sichuan Province, Sichuan Agricultural University, Chengdu, China

**Keywords:** mitogenome, *Calotes mystaceus*, phylogenetic analysis

## Abstract

A complete mitogenome sequence of the blue-crested lizard (*Calotes mystaceus*) was determined in this study. The 16,506 bp genome consists 13 protein-coding genes (PCG), two ribosomal RNA (rRNA) genes, and 22 transfer RNA (tRNA) genes, and a control region. The phylogenetic tree reveals that the *Calotes mystaceus* is closely related to the *C. versicolor*. This report provides the basic data for further studies of *Calotes* species classification and phylogeny.

The *Calotes mystaceus* belongs to the family Agamidae, genus *Calotes,* which is distributed in Indo-China, i.e. northeast India and southwest China (Yunnan Province), Myanmar, Laos, Cambodia, Vietnam and Malaysia (Saijuntha et al. 2017; Uetz et al. 2020). This species lives in the forest at mid-altitudes, as well as gardens in the lowlands and submontane forest at elevations of 180–1500 m above sea level (asl), but it also appears to be able to adapt to encroachment by humans and can be found in the tree-rich neighborhoods and city parks (Saijuntha et al. 2017). To date the mitogenome sequences of the genus *Calotes* are very limited, only the *C. versicolor* mitogenome is available, which showed a novel *tRNA-Pro* gene inversion (Amer and Kumazawa 2007). The complete mitochondrial genome of *C. mystaceus* in this study will prompt us to understand the phylogeny and molecular evolution of *Calotes*.

During the fieldwork carrying out in 2019, the specimen of *C. mystaceus* was collected from Dehong (24°22′01.20″N, 98°28′27.26″E., elev. 882 m), Yunnan Province, China. The specimen was deposited at the Museum of Sichuan Agricultural University (Specimen voucher number: 2019DH2), Chengdu, China. Total genomic DNA was extracted from ethanol-preserved muscle tissue of *C. mystaceus* by using the Ezup pillar genomic DNA extraction kit (Sangon Biotech, Shanghai, China). Then the purified DNA sample was sent to Personal Biotechnology (Shanghai, China) for library construction, sequencing, and quality control. The constructed WGS (Whole Genome Shotgun) library with approximately 400 bp insert size was sequenced using the 2 × 250 bp paired-end protocol on an Illumina MiSeq sequencing platform. To obtain high quality (HQ) clean data, a total number of 26,146,830 paired reads were cleaned up and filtered using Adapter Removal v2 and SOAPec v2.01. We used A5-miseq v20150522 and SPAdes v3.9.0 software *de novo* to assemble the obtained 24,430,798 clean reads. BLASTn was utilized to identify the contigs of the mitogenomic sequences and pilon v1.18 was selected to improve genome assembly accuracy and completeness.

The circular mitogenome (MT872513) of *C. mystaceus* is 16,506 bp long, and the gene boundaries and direction were annotated by MITOS web server. The mitogenome studied here contains 13 protein-coding genes (PCGs), two ribosomal RNA (rRNA) genes, 22 tRNA genes and a control region sequence. *Nad6* and seven *tRNA* (*tRNA-Gln, Ala, Asn, Cys, Tyr, Ser2,* and *Glu*) are located on the L-light strand, and the others (12 PCGs and 15 tRNA) are encoded on the H-heavy strand. The overall base composition of the mitogenome is 33.39%A, 13.53%G, 27.39% C and 25.68%T. Here, we also found that the *C. mystaceus* mitogenome had a *trnP* gene encoded by the heavy strand, similar gene arrangement has been found in other south and southeast Asia species of Draconinae (Amer and Kumazawa 2007; Huang et al. 2019). Eleven PCG_S_ were initiated with ATG, except two PCG_S_ (*nad2 and nad4l*) starts with ATT. Three PCG_S_ (*nad1*, *nad2* and *nad6*) were terminated by TAG as termination codon, and six (*cox1*, *cox2*, *atp8, nad4l, nad5* and *cytb*) end with TAA. *Atp6* stopped with TA, whereas three (*cox3*, *nad3* and *nad4*) end with a single base T. The length of 12S and 16S rRNA are determined to be 826 and 1,472 bp.

The nucleotide sequences of 13 PCGs were concatenated using PhyloSuite 1.2.1. We constructed the Maximum Likelihood (ML) phylogenetic tree using MEGA7.0 (Kumar et al. 2016) based on 13 concatenated PCGs. The final database using for phylogenetic analyses includes 17 sequences of 16 species from eight genera (family Agamidae), and one *Crocodylus acutus* (family Crocodylidae) was used as an outgroup. We used PartitionFinder 2.2.1 to select the nucleotide substitution model of the 13 PCGs for the ML phylogenetic tree construction. The topology of phylogenetic trees revealed that the newly sequenced *C. mystaceus* clusters with *C. versicolor* with 100% bootstrap values (Figure 1). The complete mitochondrial genome of *C. mystaceus* in this study will prompt us to understand the phylogeny and molecular evolution of *Calotes*.

**Figure 1.**
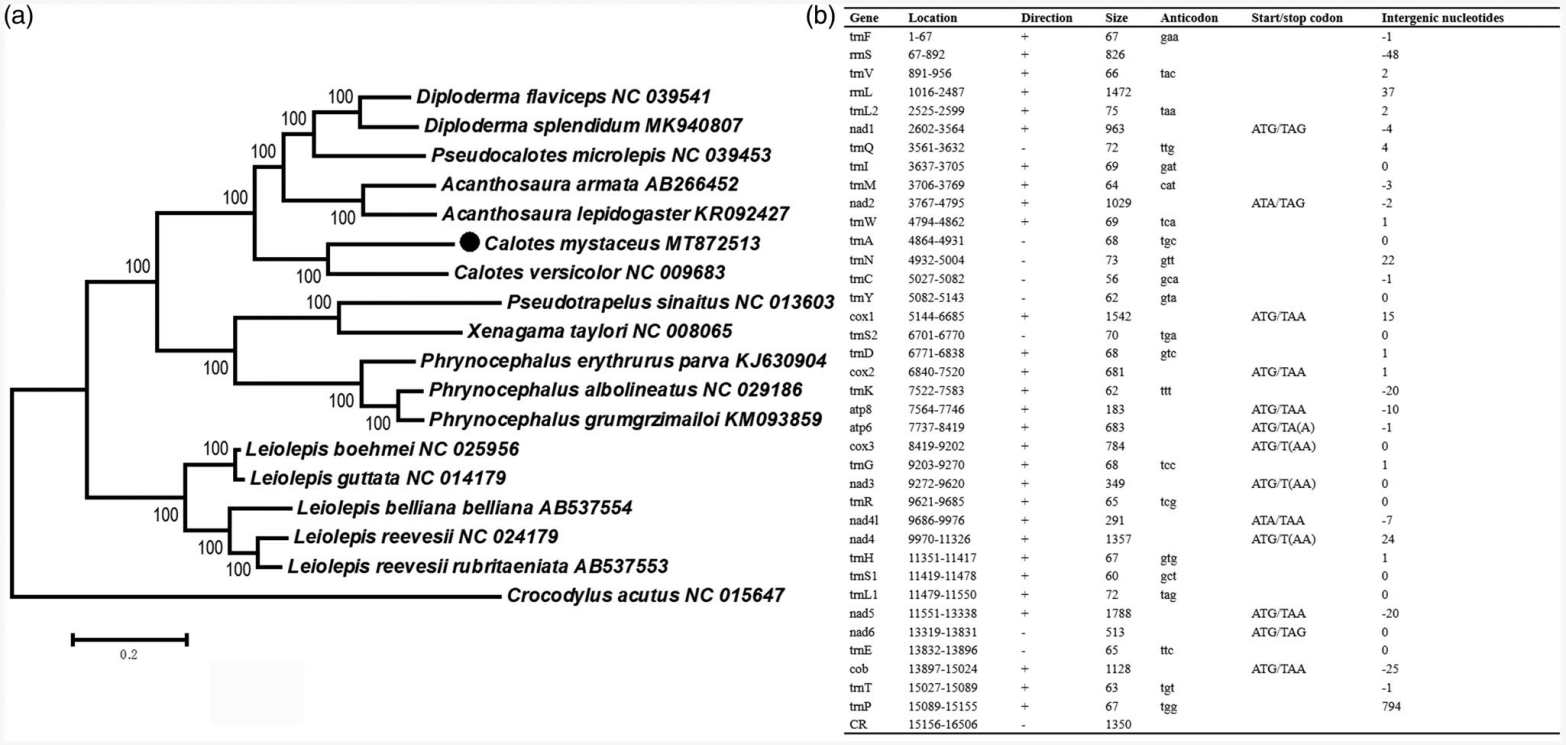
Phylogenetic tree and annotation of the *C. mystaceus* mitogenome. (a) Maximum-likelihood (ML) phylogenetic tree of twenty-one reptile species based on 13 protein-coding genes. Bootstrap support values are shown at the node. The GenBank accession numbers are listed following species names. (b) annotation of the complete mitogenome of *Calotes mystaceus.*

## Data Availability

The data used to support the findings of this study are available in GenBank at https://www.ncbi.nlm.nih.gov/nuccore/MT872513, reference number MT872513.
